# Possible mechanisms involved in the anti-nociceptive effects of hydro-ethanolic leaf extract of *Ziziphus abyssinica*

**DOI:** 10.1080/13880209.2017.1355927

**Published:** 2017-07-20

**Authors:** Eric Boakye-Gyasi, Isaac Tabiri Henneh, Wonder Kofi Mensah Abotsi, Elvis Ofori Ameyaw, Eric Woode

**Affiliations:** a Department of Pharmacology, Faculty of Pharmacy and Pharmaceutical Sciences, Kwame Nkrumah University of Science and Technology, Kumasi, Ghana;; b Department of Pharmacology, School of Medical Sciences, University of Cape Coast, Cape Coast, Ghana;; c Department of Biomedical Sciences, School of Allied Health Sciences, University of Cape Coast, Cape Coast, Ghana

**Keywords:** TNF-α, IL-1β, prostaglandin, bradykinin, nociception

## Abstract

**Context:** Various parts of *Ziziphus abyssinica* Hochst ex. A. Rich (Rhamnaceae) have been used in Ghanaian and African traditional medicine as an analgesic. However, there are little scientific data to support the anti-nociceptive effects of the hydro-ethanolic leaf extract of *Ziziphus abyssinica* (EthE) as well as the possible mechanisms involved in its anti-nociceptive effects.

**Purpose:** To predict possible nociceptive pathways involved in the anti-nociceptive effects of EthE.

**Materials and methods:** The effect of EthE (30, 100 and 300 mg/kg) on intraplantar injection of pain mediators such as interleukin-1β, tumour necrosis factor-α, prostaglandin E_2_ and bradykinin was evaluated in male Sprague Dawley rats using Randall–Selitto test for 5 h. The effect of specific antagonists to the opioidergic, adenosinergic, ATP-sensitive K^+^ channels, nitric oxide, serotonergic, muscarinic, adrenergic and voltage-gated calcium channel on the anti-nociceptive effect of EthE (100 mg/kg) was evaluated using the formalin test in male imprinting control region (ICR) mice for 1 h.

**Results:** Pretreatment of the rats with EthE significantly reversed the hypernociception induced by intraplantar injection of TNF-α (*F*_4,120_ = 10.86, *p* < 0.0001), IL-1β (*F*_4,120_ = 14.71, *p < *0.0001), bradykinin (*F*_4,80_ = 12.52, *p < *0.0001) and prostaglandin E_2_ (*F*_5,144_ = 6.165, *p =* 0.0001). The anti-nociceptive effect exhibited by EthE in the formalin test was reversed by systemic administration of N^G^-l-nitro-arginine methyl ester, naloxone, theophylline and glibenclamide.

**Conclusions:** EthE inhibits hypernociception induced by TNF-α, IL-1β, bradykinin and prostaglandin E_2_. EthE exhibited anti-nociceptive effects possibly mediated through opioidergic, adenosinergic, ATP-sensitive potassium channels and nitric oxide cyclic GMP pathways.

## Introduction

Pain is a personal and subjective experience that involves emotional, behavioural and sensory elements associated with actual or potential tissue damage (Merskey and Bugduk 1994). It is a direct or indirect consequence of several diseases and is the commonest reason for hospital visits (American Pain Society 2000). In both developing and developed countries, patients with moderate to severe pain are often under-treated because opioids, which are the mainstay of pain relief in such cases, are mostly inaccessible. This is a result of the fact that opioids are categorized as controlled substances and therefore are subjected to stringent control (Kumar 2007).

Inflammatory mediators such as prostaglandins and bradykinins are marked sensitizers of nociceptors. These mediators facilitate the electrical activity of the neuronal membrane by acting on neuronal receptors directly resulting in the activation of several molecular mechanisms which subsequently causes hypernociception (Linley et al. 2010; Schaible et al. 2011).

Cytokines are important mediators of peripheral sensitization. Intradermal administration of cytokines such as keratinocyte-derived chemokine (KC), TNF-α, interleukins IL-1β, IL-8, IL-12, IL-15 and IL-18 have produced intense and sustained mechanical sensitization and hypernociception in rodents (Stein et al. 2009). It has been proposed that TNF-α can be stimulated by irritants such as carrageenan, lipopolysaccharide or the antigen itself which, in turn, induces IL-1β and IL-6, thus activating the synthesis of cyclooxygenase products (PGE_2_). Again, TNF-α can induce another cytokine, IL-8, thus stimulating the local production of sympathetic amines which subsequently produces hypernociception. Also, endothelin-1 can be activated by IL-18 and IL-12 resulting in hypernociception (Ferreira et al. 1993).

Understanding the exact mechanism of medicinal substances and blocking or antagonizing these pathways have been the focus of researchers in recent times and have resulted in the development of novel analgesics. Compounds such as TRPV1 antagonists (Willis 2009), nerve growth factor (NGF) antagonists (Watson et al. 2008; Cattaneo 2010), selective Na channel blockers (Jarvis et al. 2007), anti-TNF-α and interleukin-1 monoclonal antibodies (Furst et al. 2003; Vale et al. 2004), and bradykinin receptor antagonists (Rodger 2009) have been targeted in the search for novel analgesics to augment or replace the already available analgesics.

Plants also constitute a large source of novel phytocompounds that might lead to the discovery of new pharmaceutical agents (Shah and Alagawadi 2011). About 25% of drugs prescribed worldwide are derived from plants and their use as medicines is still widespread (Wachtel-Galor and Benzie 2011). One such plant is *Ziziphus abyssinica* Hochst Ex A. Rich (Rhamnaceae), commonly called ‘catch thorn’ in English and ‘Jujubier sauvage’ in French; the Hausa’s call it ‘magariya’, whereas in Ghana it is called ‘larukluror’ (Sisaala) (Burkill 1985; Orwa et al. 2009). Extracts from various parts of the plant have exhibited antioxidant, antibacterial and antifungal activities (Gundidza and Sibanda 1991; Nyaberi et al. 2010; Wagate et al. 2010). Molluscicidal (Kela et al. 1989) and antiplasmodial (Muthaura et al. 2015) activities of the plant have been reported. Root extracts have been reported to possess anti-ulcerogenic (Ugwah et al. 2013) and antidiarrheal (Ugwah-Oguejiofor et al. 2013) properties. Our earlier research validated the analgesic effect of the hydro-ethanolic leaf extract of the plant in murine models (Boakye-Gyasi et al. 2017). Qualitative phytochemical investigations have revealed that the aqueous and methanol fruit extracts of *Z. abyssinica* contain saponins, tannins, sterols and steroids, alkaloids, flavonoids and reducing compounds (Nyaberi et al. 2010). Also the presence of carbohydrates, alkaloids, saponins, tannins, glycosides, anthraquinones and steroids were detected in *Z. abyssinica* aqueous root extract (Ugwah et al. 2013). Hydro-ethanolic leaf extract of the plant has been reported to contain tannins, phenols, alkaloids, triterpenes, flavonoids and phytosterols (Boakye-Gyasi et al. 2017).

Since there is no report on the possible mechanisms mediating the anti-nociceptive effects of the hydro-ethanolic leaf extract of *Ziziphus abyssinica*, this study seeks to determine the possible involvement of some important inflammatory mediators, cytokines, ion channels, receptors and ligands in the anti-nociceptive effect of the extract.

## Method and materials

### Plant collection

Fresh leaves of *Ziziphus abyssinica* were collected from Ejura (7^°^23′00.16″N, 1^°^22′00.00″W) in the Ejura-Sekyedumase Municipal of Ashanti Region in the month of October 2015. It was authenticated by Mr. Clifford Asare of the Department of Herbal Medicine, Faculty of Pharmacy and Pharmaceutical Sciences (FPPS), Kwame Nkrumah University of Science and Technology (KNUST). A voucher specimen (KNUST/HM/2016/L003) was deposited at the Department of Herbal Medicine’s herbarium.

### Plant extraction

About 1 kg of fresh matured leaves of *Ziziphus abyssinica* was air-dried for 14 days in a room. With the aid of a hammer mill, it was pulverized into fine powder. The powdered leaves (600 g) were extracted with 4 L of 70% v/v ethanol for 48-h period using a Soxhlet apparatus (Aldrich^®^ Soxhlet Extraction Apparatus, Z556203, St. Louis, MO). The extract obtained was labelled as EthE (hydro-ethanolic extract) and subsequently concentrated using a rotary evaporator (Rotavapor R-215 model, BÜCHI Labortechnik AG, Flawil, Switzerland) under reduced pressure and temperature (70 °C). This was further dried on a water bath and then preserved in a desiccator containing activated silica until it was ready for use. The yield obtained was 10.8% w/w.

### Animals

Male imprinting control region (ICR) mice (20–25 g) and Sprague Dawley rats (170–250 g) were bought from Noguchi Memorial Institute for Medical Research, University of Ghana, Legon, Ghana. They were kept in stainless cages (34 cm × 47 cm × 18 cm) in groups of five at the animal house facility of Faculty of Pharmacy and Pharmaceutical Sciences (FPPS), KNUST, Kumasi. The animals were given normal commercial diet obtained from Agricare Limited, Kumasi, Ghana, and water *ad libitum*. They were kept under normal laboratory conditions with regard to room temperature and humidity. All the techniques and protocols used in the study were done in accordance with established public health guidelines in ‘Guide for Care and Use of Laboratory Animals’ (Garber et al. 2011). Also, all protocols used in the study were approved by the Department of Pharmacology, FPPS, KNUST Ethics Committee.

### Drugs and chemicals

The following chemicals and drugs were used in the study: formalin, theophylline, carrageenan and acetic acid (British Drug House, Poole, England); granisetron hydrochloride (Corepharma LLC, Middlesex, NJ); glibenclamide (Daonil^®^, Sanofi-Aventis, Guildford, UK); yohimbine hydrochloride (Procomil^®^, Walter Ritter GmbH & Co. KG, Hamburg, Germany); atropine sulphate, naloxone hydrochloride, morphine sulphate (Duopharma (M) Sdn Bhd, Malaysia); nifedipine and diclofenac sodium (Denk Pharma, Munich, Germany); and l-glutamic acid, N^G^-l-nitro-arginine methyl ester (L-NAME), prostaglandin E_2_ (PGE_2_), bradykinin acetate salt, murine recombinant interleukin-1β (IL-1β) and tumour necrosis factor-alpha (TNF-α) (Sigma-Aldrich Inc., St. Louis, MO).

### Tumour necrosis factor-alpha (TNF-α)-induced hypernociception

Assessment of mechanical hypernociception induced by TNF-α after pretreatment of rats with EthE or morphine was performed as previously described by Vale et al. (2004). Five groups of rats (*n* = 5) received pretreatment with either vehicle (10 mL/kg, p.o.), EthE (30, 100 and 300 mg/kg p.o.) for 1 h or morphine (3 mg/kg, i.p.) for 30 min before intraplantar injection of TNF-α (2.5 pg/paw; 20 μL) into the right hind paw. Hypernociception was measured in the injected paws at 1, 2, 3, 4 and 5 h post-TNF-α injection using an analgesimeter (Model No. 15776, Ugo Basile, Comerio, Varese, Italy) as described previously (Randall and Selitto 1957; Villetti et al. 2003). The rats were trained at three different times before the day of testing and it involved gradually applying pressure to their right hind paws. The applied pressure (grams) able to elicit paw withdrawal was recorded as paw withdrawal threshold (PWT). A cut-off threshold of 250 g was set in order not to cause any injury to the paws. Baseline thresholds were taken for each animal before they were given TNF-α.

Percentage maximum possible effect was determined using the formula below:

$$\%MPE=\left(\frac{PWT-CT}{250g-CT}\right)\times 500$$

This same procedure was used to measure hypernociception in the IL-1β, PGE_2_ and bradykinin-induced hypernociceptions below.

### Interleukin-1-beta (IL-1β)-induced hypernociception

Assessment of mechanical hypernociception induced by IL-1β after pretreatment of rats with EthE or morphine was performed as previously described by Vale et al. (2004). Five groups of male rats (*n* = 5) received pretreatment with either vehicle (10 mL/kg, p.o.), EthE (30, 100 and 300 mg/kg, p.o.) for 1 h or morphine (3 mg/kg, i.p.) for 30 min before intraplantar injection of IL-1β (1 pg/paw; 20 μL) into the right hind paws.

### Prostaglandin E_2_ (PGE_2_)-induced hypernociception

The effect of pretreatment of rats with EthE on prostaglandin E_2_-induced hypernociception was evaluated as earlier described (Vale et al. 2004; Woode et al. 2012). Six groups of rats (*n* = 5) received pretreatment with either vehicle (10 mL/kg, p.o.) or EthE (30–300 mg/kg, p.o.) for 1 h and morphine (3 mg/kg, i.p.) or diclofenac (10 mg/kg, i.p.) for 30 min before intraplantar injection of PGE_2_ (100 ng/paw; 20 μL) into their right hind paws.

### Bradykinin-induced hypernociception

To evaluate the effect of EthE pretreatment on mechanical hypernociception induced by bradykinin in rats, a previously described method was used (Vale et al. 2004; Woode et al. 2012). Five groups of rats (*n* = 5) were pretreated with either EthE (30, 100 and 300 mg/kg, p.o., 1 h) or morphine (3 mg/kg, i.p., 30 min) before intraplantar injection of bradykinin (500 ng/paw; 20 μL) into their right hind paws. The control group received vehicle (10 mL/kg, p.o., 1 h). Rats were pretreated with captopril (5 mg/kg, s.c.) 1 h before the experiment to avoid break down of bradykinin by angiotensin converting enzyme.

### Further investigation into the possible mechanism of EthE anti-nociceptive effect

Formalin test (Tjølsen et al. 1992; Ellis et al. 2008) was used to further assess the possible involvement of the opioidergic, adenosinergic, ATP-sensitive K^+^ channels, nitric oxide, serotonergic, muscarinic, adrenergic and voltage-gated calcium channel pathways in the observed anti-nociceptive effect of the extract. Doses of drugs were selected based on preliminary experiments in our laboratories and previous studies (Woode et al. 2012).

To investigate the roles played by these nociceptive pathways, nineteen groups of mice (*n* = 5) were pretreated orally with naloxone (2 mg/kg, i.p., a nonselective opioid receptor antagonist), theophylline (5 mg/kg, p.o., a nonselective adenosine receptor antagonist), glibenclamide (8 mg/kg, p.o., an ATP-sensitive K^+^ channel inhibitor), L-NAME, (10 mg/kg, i.p., a nitric oxide synthase inhibitor), granisetron (2 mg/kg, p.o., a 5HT_3_ receptor antagonist), atropine (3 mg/kg, i.p., nonselective muscarinic antagonist), yohimbine (3 mg/kg, p.o., an α_2_ receptor antagonist) and nifedipine (10 mg/kg, p.o., L-type voltage-gated calcium channel blocker). After 60 min (p.o.) or 30 min (i.p.) pretreatment with various antagonists, mice were either given oral administration of 100 mg/kg EthE or intraperitoneal injection of 3 mg/kg morphine. Negative control group received only vehicle (10 mL/kg, p.o.), whereas EthE- and morphine-treated controls received either 100 mg/kg EthE or 3 mg/kg morphine only. After one-h EthE or 30-min morphine treatments, pain was induced with 10 μL of 5% formalin in all the groups and nociceptive score was measured for 1 h. To do this, the mice were instantly transferred into transparent testing perspex chambers (15 cm ×15 cm ×15 cm). A mirror placed at 45° to the floor level allowed complete view of the animals in the camcorder (Sony Handycam, model HDRCX675/B, Tokyo, Japan) which was used to capture the nociceptive behaviours of the mice following formalin injection. This was recorded for 60 min and later tracked using a JWatcher^™^ software version 1.0 developed by University of California, Los Angeles, CA, USA and Macquarie University, Sydney, Australia (http://www.jwatcher.ucla.edu/). A nociceptive score for every 5 min time bloc was obtained by measuring the duration and frequency of licking/biting of injected paws and the mean nociceptive score for each time bloc per 5 min determined as the product of the duration and frequency of licking/biting. The results obtained were considered as early/neurogenic phase (0–10 min) and late/inflammatory phase (10–60 min) from which time-course curves were plotted and the areas under the curves (AUCs) for each phase and each treatment determined and plotted.

### Acute toxicity study

ICR mice (25–30 g) were randomly selected and divided into seven groups of five mice in each group. They were fasted overnight and given EthE (30, 100, 300, 1000, 3000 and 5000 mg/kg, p.o.). The control group received 10 mL/kg p.o. of normal saline. The mice were observed at time 0, 30, 60, 120 and 180 min after drug administration for any toxic signs or death. They were also observed at 24 h and daily for up to 7 days to detect any possible delayed deaths.

### Statistical analysis

A sample size of five rats or mice per group was used in all *in vivo* tests. Mean ± standard error of mean (SEM) was used in presenting all data. All time-course curves in the study were analyzed using two-way analysis of variance (ANOVA) with Bonferroni’s *post hoc* test. One-way ANOVA with Newman–Keuls *post hoc* test was used to determine differences between treatments groups (areas under the curves). The equation below was used to calculate the percentage inhibition for each treatment:

$$\%inhibition=\left(\frac{AUC_{control}-AUC_{treatment}}{AUC_{control}}\right)\times 100$$

GraphPad^®^ Prism version 7.0 (GraphPad Software, San Diego, CA) for Windows was used to perform all statistical analysis with *p* < 0.05 considered statistically significant for all tests.

## Results

### TNF-α-induced hypernociception

**Figure 1.**
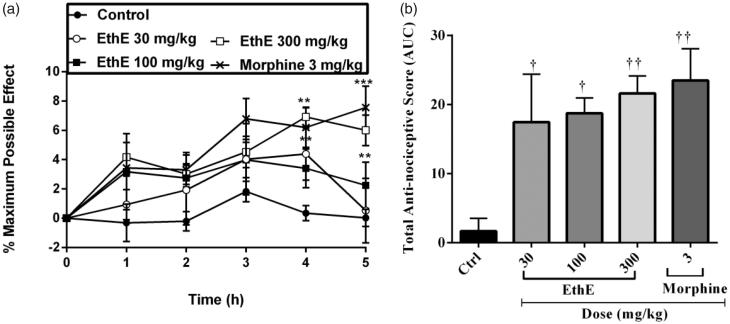
Effect of pretreatment of rats with EthE (30–300 mg/kg, p.o.) and morphine (3 mg/kg, i.p.) on TNF-α-induced hypernociception. Each datum represents the mean of five animals and the error bars indicate SEM. The symbols * and **†** indicate significance levels compared to respective control groups: (a) represent the time-course curves ***p < 0.001, **p < 0.01 (two-way ANOVA followed by Bonferroni’s *post hoc* test), whereas (b) represents total anti-nociceptive effects (AUC) **††***p <* 0.01 and **†***p <* 0.05 (one-way ANOVA followed by Newman–Keuls *post hoc* test).

The results presented in Figure 1(a,b) show that EthE and morphine markedly reversed hypernociception induced by intraplantar injection of TNF-α irritant. Pretreatment of the rats with EthE (30, 100 and 300 mg/kg, p.o.) and morphine (3 mg/kg, i.p.) led to a significant decrease in the paw withdrawal latencies of rats (*F*_4,120_ = 10.86, *p* < 0.0001, Figure 1(a)) and total anti-nociceptive score (*F*_4,18_ = 5.811, *p* = 0.005, Figure 1(b)). The highest dose of EthE completely reversed TNF-α-induced mechanical hypernociception with an average total anti-nociceptive score of 21.62 ± 2.52, whereas morphine (3 mg/kg, i.p.) similarly reversed the hypernociception with an average total anti-nociceptive score of 23.49 ± 4.59 (Figure 1(a,b)).

### Interleukin-1β-induced hypernociception

**Figure 2.**
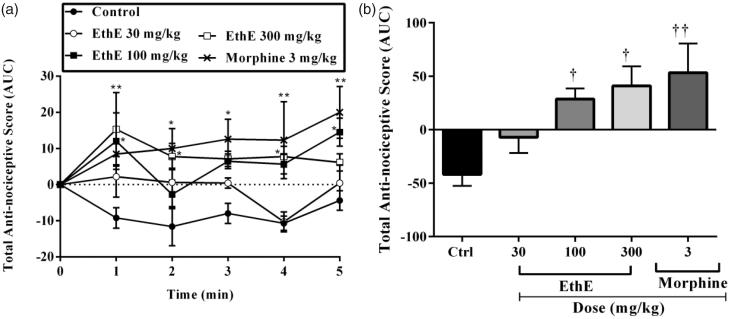
Effect of pretreatment of rats with EthE (30–300 mg/kg, p.o.) and morphine (3 mg/kg, i.p.) on IL-1β-induced hypernociception. Each datum represents the mean of five animals and the error bars indicate SEM. The symbols * and † indicate significance levels compared to respective control groups: (a) represents the time-course curves **p < 0.01, *p < 0.05 (two-way ANOVA followed by Bonferroni’s *post hoc* test), whereas (b) represents total anti-nociceptive effects (AUC) **††***p <* 0.01 and **†***p <* 0.05 (one-way ANOVA followed by Newman–Keuls *post hoc* test).

The results presented in Figure 2(a,b) show that intraplantar injection of IL-1β prominently decreased rats’ paw withdrawal thresholds in the Randall–Selitto test compared to baseline readings and this has been described as a state of hypernociception which was maintained throughout the test in the vehicle-treated rats. Pretreatment of the animals with either EthE (30, 100 or 300 mg/kg, p.o.) or morphine (3 mg/kg, i.p.) significantly reversed the hypernociception by increasing paw withdrawal thresholds (*F*_4,120_ = 14.71, *p <* 0.0001, Figure 2(a)) and total anti-nociceptive score (*F*_4,20_ = 5.96, *p* = 0.0025, Figure 2(b)). From the results presented in Figure 2(b), EthE (300 mg/kg, p.o.) completely reversed IL-1β-induced hypernociception with a mean total anti-nociceptive score of 40.99 ± 18.27 even though it was less potent than morphine (3 mg/kg, i.p.) which had a mean total anti-nociceptive score of 65.33 ± 26.91 (Figure 2(a,b)).

### Bradykinin-induced hypernociception

**Figure 3.**
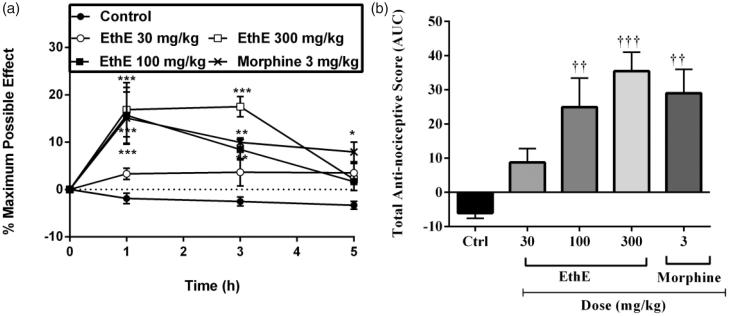
Effect of pretreatment of rats with EthE (30–300 mg/kg, p.o.) and morphine (3 mg/kg, i.p.) on bradykinin-induced hypernociception. Each datum represents the mean of five animals and the error bars indicate SEM. The symbols * and **†** indicate significance levels compared to respective control groups: (a) represents the time-course curves ***p < 0.001, **p < 0.01, *p < 0.05 (two-way ANOVA followed by Bonferroni’s *post hoc* test), whereas (b) represents total anti-nociceptive effects (AUC) **†††***p <* 0.001 and **††***p <* 0.01 (one-way ANOVA followed by Newman–Keuls *post hoc* test).

Mechanical pressure applied to the rats’ right hind paws after intraplantar injection of bradykinin resulted in an increase in paw withdrawal reflexes which can be described as a state of hypernociception in the animals. Pretreatment of the animals with either EthE (30, 100 or 300 mg/kg, p.o.) or morphine (3 mg/kg, i.p.) led to a significant increase in paw withdrawal latencies (*F*_4,80_ = 12.52, *p <* 0.0001, Figure 3(a)) and total anti-nociceptive score (*F*_4,20_ = 8.353, *p* = 0.0004, Figure 3(b)). The highest dose of EthE produced a mean total anti-nociceptive score of 35.45 ± 5.55, whereas morphine (3 mg/kg, i.p.) produced a total anti-nociceptive score of 29.0 ± 6.96 (Figure 3).

### Prostaglandin E_2_-induced hypernociception

**Figure 4.**
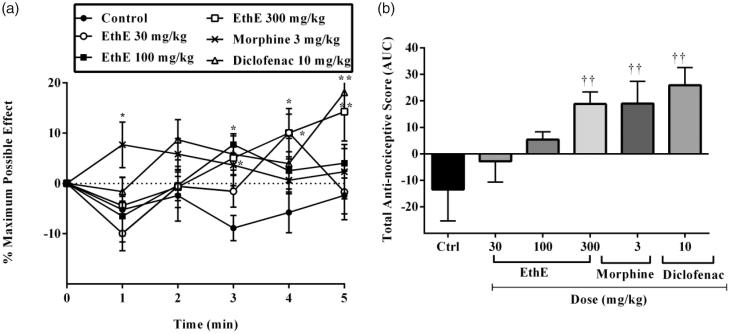
Effect of pretreatment of rats with EthE (30–300 mg/kg, p.o.), morphine (3 mg/kg, i.p.) and diclofenac (10 mg/kg, i.p.) on PGE_2_-induced hypernociception. Each datum represents the mean of five animals and the error bars indicate SEM. The symbols * and **†** indicate significance levels compared to respective control groups: (a) represents the time-course curves **p < 0.01, *p < 0.05 (two-way ANOVA followed by Bonferroni’s *post hoc* test), whereas (b) represents total anti-nociceptive effects (AUC) **††***p <* 0.01 (one-way ANOVA followed by Newman–Keuls *post hoc* test).

Intraplantar administration of prostaglandin E_2_ irritant (100 ng/paw, 20 μL) resulted in a decrease in paw withdrawal threshold upon the application of mechanical pressure to the right hind paws of the rats and this can be described as a state of hypernociception. Pretreatment of the rats with EthE (30, 100 and 300 mg/kg, p.o.), morphine (3 mg/kg, i.p.) and diclofenac (10 mg/kg, i.p.) resulted in a significant increase in paw withdrawal thresholds (*F*_5,144_ = 6.165, *p =* 0.0001, Figure 4(a)) and total anti-nociceptive scores (*F*_5,24_ = 5.811, *p* = 0.0012, Figure 4(b)). The highest dose of EthE completely reversed the hypernociception induced by PGE_2_ with a mean total anti-nociceptive score of 17.31 ± 5.84. Morphine (3 mg/kg, i.p.) and diclofenac (10 mg/kg, i.p.) similarly reversed the hypernociception with mean total anti-nociceptive scores of 18.98 ± 8.40 and 25.9 ± 6.66, respectively (Figure 4(a,b)).

### Further assessment of possible mechanism of action of EthE

**Figure 5.**
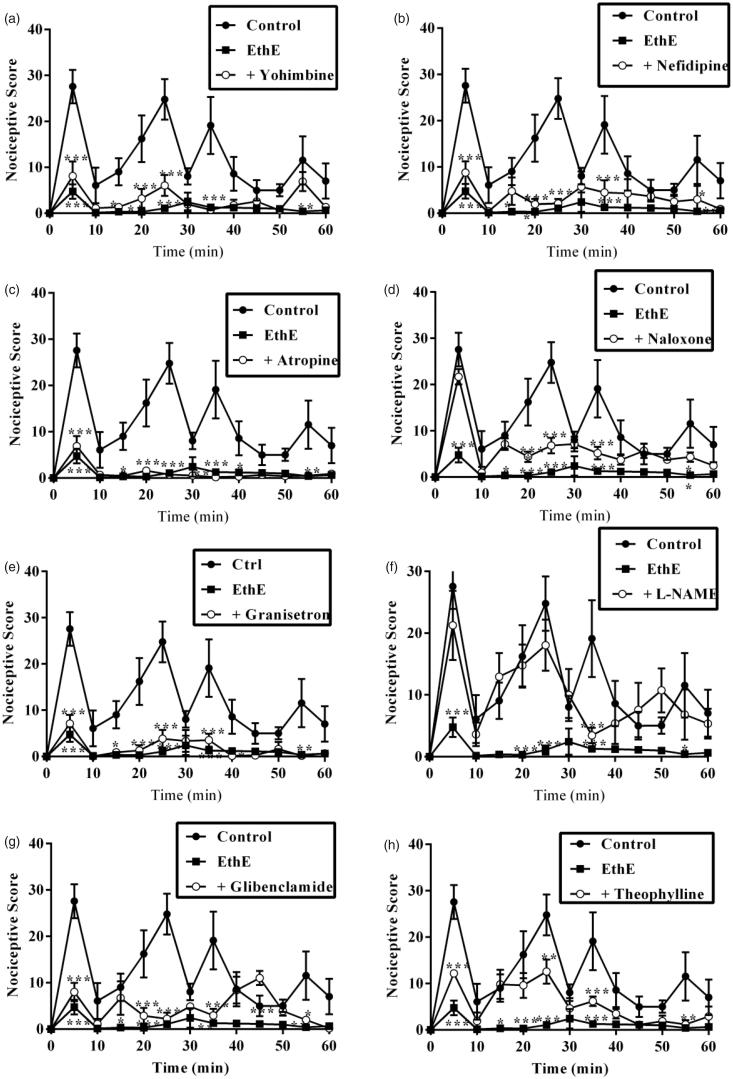
Effect of pretreatment of mice with (a) yohimbine (3 mg/kg, p.o.), (b) nifedipine (10 mg/kg, p.o.), (c) atropine (5 mg/kg, i.p.), (d) naloxone (2 mg/kg, i.p.), (e) granisetron (2 mg/kg, p.o.), (f) L-NAME (10 mg/kg, i.p.), (g) glibenclamide (8 mg/kg, p.o.) and (h) theophylline (10 mg/kg, i.p.) on the nociceptive scores of EthE (100 mg/kg, p.o.) on the time-course curves of formalin-induced nociceptive test. Each point represents the mean of five animals and the error bars indicate SEM. ***p < 0.001, **p < 0.01 and *p < 0.05 compared to the control group at same time points (two-way ANOVA followed by Bonferroni’s *post hoc* test).

**Figure 6.**
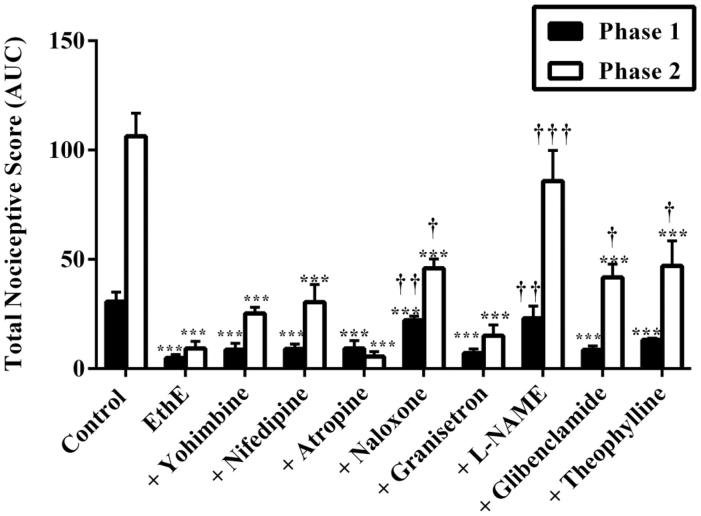
Effect of pretreatment of mice with yohimbine (3 mg/kg, p.o.), nifedipine (10 mg/kg, p.o.), atropine (5 mg/kg, i.p.), naloxone (2 mg/kg i.p.), granisetron (2 mg/kg, p.o.), L-NAME (10 mg/kg, i.p), glibenclamide (8 mg/kg, p.o.) and theophylline (10 mg/kg, i.p.) on the total nociceptive score of EthE (100 mg/kg, p.o.) in phase 1 and phase 2 of formalin-induced nociception. Each column represents the mean of five animals and the error bars indicate SEM. ***p < 0.001 compared to control group and **†††***p <* 0.001, **††***p <* 0.01 and **†***p <* 0.05 compared to EthE-alone-treated group (one-way ANOVA followed by Newman–Keuls *post hoc* test).

Results presented in Figures 5 and 6 show the effect of pretreatment of mice with yohimbine (3 mg/kg, p.o.), nifedipine (10 mg/kg, p.o.), atropine (5 mg/kg, i.p.), naloxone (2 mg/kg, i.p.), granisetron (2 mg/kg, p.o.), L-NAME (10 mg/kg, i.p.), glibenclamide (8 mg/kg, p.o.) and theophylline (10 mg/kg, i.p.) on the anti-nociceptive effects of EthE. Both phase 1 and phase 2 anti-nociceptive effects of EthE (100 mg/kg, p.o.) were significantly reversed by pretreatment of mice with naloxone and L-NAME. Pretreatment of mice with glibenclamide and theophylline significantly reversed the anti-nociceptive effect of EthE in only the second phase of formalin test. Yohimbine, nifedipine, atropine and granisetron could not significantly reverse EthE anti-nociception in both phases of formalin test (Figures 5 and 6).

**Figure 7.**
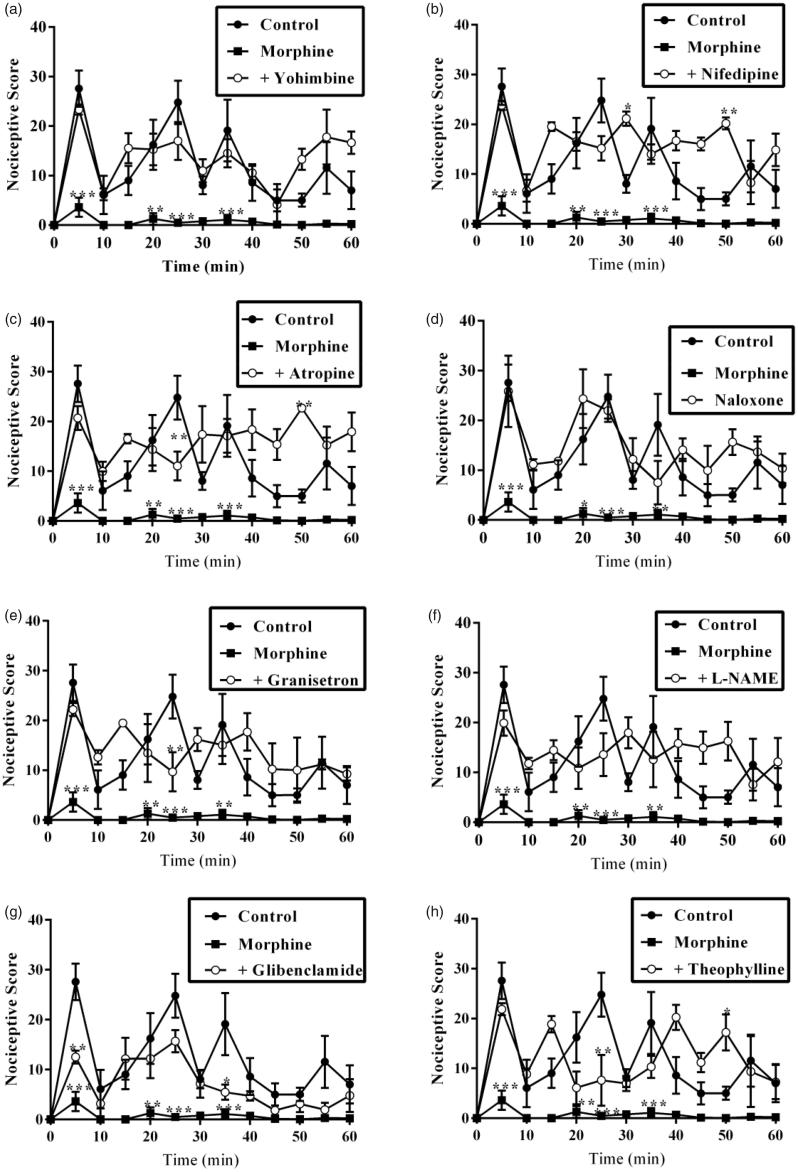
Effect of pretreatment of mice with (a) yohimbine (3 mg/kg, p.o.), (b) nifedipine (10 mg/kg, p.o.), (c) atropine (5 mg/kg, i.p.), (d) naloxone (2 mg/kg, i.p.), (e) granisetron (2 mg/kg, p.o.), (f) L-NAME (10 mg/kg, i.p.), (g) glibenclamide (8 mg/kg, p.o.) and (h) theophylline (10 mg/kg i.p.) on the total nociceptive score of morphine (3 mg/kg, i.p.) on the time-course curves of formalin-induced nociceptive test. Each point represents the mean of five animals and the error bars indicate SEM. ***p < 0.001, **p < 0.01 and *p < 0.05 compared the control group at same time points (two-way ANOVA followed by Bonferroni’s *post hoc* test).

**Figure 8.**
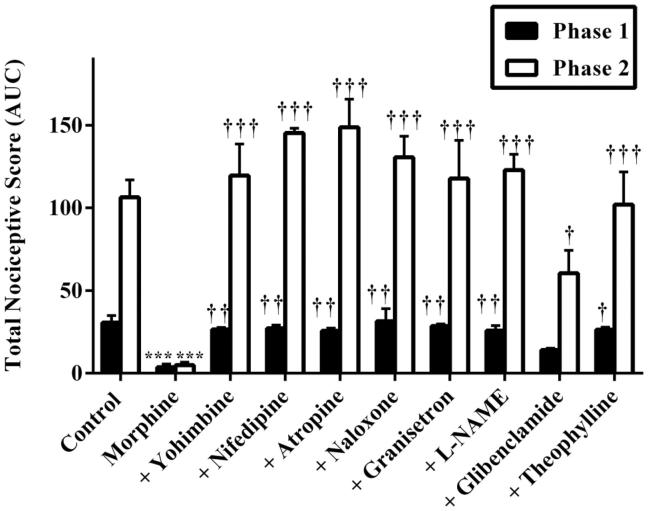
Effect of pretreatment of mice with yohimbine (3 mg/kg, p.o.), nifedipine (10 mg/kg, p.o.), atropine (5 mg/kg, i.p.), naloxone (2 mg/kg i.p.), granisetron (2 mg/kg, p.o.), L-NAME (10 mg/kg, i.p), glibenclamide (8 mg/kg, p.o.) and theophylline (10 mg/kg, i.p.) on the total nociceptive scores of morphine (3 mg/kg, i.p.) in phase 1 and phase 2 of formalin-induced nociception. Each column represents the mean of five animals and the error bars indicate SEM. ***p < 0.001 compared to control group and **†††***p <* 0.001, **††***p <* 0.01 and **†***p ≤* 0.05 compared to morphine-alone-treated group (one-way ANOVA followed by Newman–Keuls *post hoc* test).

Also, results presented in Figures 7 and 8 show the effect of pretreatment of mice with yohimbine (3 mg/kg, p.o.), nifedipine (10 mg/kg, p.o.), atropine (5 mg/kg, i.p.), naloxone (2 mg/kg, i.p.), granisetron (2 mg/kg, p.o.), L-NAME (10 mg/kg, i.p.), glibenclamide (8 mg/kg, p.o.) and theophylline (10 mg/kg, i.p.) on the anti-nociceptive effect induced by morphine (3 mg/kg, i.p.). Intraperitoneal administration of morphine (3 mg/kg, i.p.) resulted in a significant decrease in total nociception induced by intraplantar injection of 5% formalin (*p* < 0.001). The anti-nociceptive effect of morphine (3 mg/kg, i.p.) was markedly reversed by pretreatment of mice with all the antagonists used in both phases except glibenclamide. Pretreatment of mice with glibenclamide (8 mg/kg, p.o.) 30 min before morphine (3 mg/kg, i.p.) significantly (*p* < 0.05) reversed the anti-nociceptive effect of the latter in only the inflammatory phase but not the neurogenic phase (Figures 7 and 8).

### Acute toxicity study

There was no lethality recorded during the 7-day study period at doses up to 5000 mg/kg. Treated mice did not exhibit any toxic signs in terms of their behavioural, neurological and autonomic activities as compared to control.

## Discussion

Intraplantar injection of TNF-α antigen is known to stimulate IL-1β production, thus inducing the production of cyclooxygenase products such as prostaglandin E_2_ which subsequently causes hypernociception (Verri et al. 2007). A common feature of the inflammatory response produced by these pro-inflammatory cytokines (TNF-α and IL-1β) when injected intraplantarly is increased pain sensitivity which occurs as a result of their direct action on their respective targets, thus decreasing pain thresholds resulting in hypernociception (Jin and Gereau 2006; Binshtok et al. 2008). EthE significantly reversed hypernociception induced by intraplantar injection of TNF-α and IL-1β, suggesting a possible blockade of their targets or inhibitory effect on their release peripherally and/or centrally.

Similarly, the hypernociception induced by prostaglandin E_2_ and bradykinin, marked sensitizers of nociceptors, was both reversed significantly and dose dependently by the extract EthE. By binding to G protein-coupled receptors, prostaglandin E_2_ increases cAMP which subsequently activates PKA in cells. This pathway enhances excitability of neurons by sensitizing ion channels in membranes such as TRPV1 receptors and Na^+^ channels (Schaible et al. 2011). On the other hand, the mechanical hypernociception induced by bradykinin involves an indirect activation of PKA and a direct activation of B_2_ receptor-mediated phospholipase C (PLC) which in turn leads to the production of PKC resulting in the sensitization of sensory ion channels which subsequently leads to hypernociception (Ferreira et al. 2004; Linley et al. 2010). EthE might have acted possibly by inhibiting the release or interfering with some of the pathways of these mediators, thus reversing the hypernociception.

In an attempt to further identify other pathways possibly involved in the pain-relieving effect of extract, mice were treated with either EthE or morphine in the presence of the following antagonists: yohimbine, nifedipine, atropine, naloxone, granisetron, L-NAME, glibenclamide and theophylline. The ability of the antagonists to reverse the pain-relieving activity of the extract or morphine was then assessed in the formalin-induced nociception test.

The anti-nociceptive effect of EthE was markedly overturned in both phases of the formalin test following pretreatment of animals with L-NAME, a nitric oxide (NO) synthase inhibitor. The findings implicate the involvement of the NO cGMP mechanism. NO is a constantly synthesized soluble gas from L-arginine amino acid in endothelial cells by the nitric oxide synthase (NOS) enzyme (Mayer and Hemmens 1997). Nitric oxide is known to play a complex part in transmission of nociceptive signals peripherally and centrally (Cury et al. 2011; Galdino et al. 2015). Nitric oxide is known to be involved in the inhibition of neurons that are spontaneously activated in response to pain and particularly diminishing the activity of pain mediators such as substance P released in spinal cord (Garry et al. 1994; Schmid and Pehl 1996). The marked reversal of EthE anti-nociceptive activity by NOS antagonist, L-NAME, is an indication of a possible involvement of NO cGMP pathway in the extract’s mode of anti-nociception action.

Furthermore, pretreatment with naloxone, a nonselective opioid antagonist, also blocked the anti-nociceptive effects of EthE which might implicate the involvement of opioid receptors and/or endogenous opioids in the anti-nociceptive effects of the extract (Bovill 1997; Kiran and Sinha 2015).

Adenosine is an important endogenous modulator of neurotransmission and it participates in modulating several biological activities including nociception. Such effects are mediated through the stimulation of its G protein-coupled receptors A_1_, A_2a_, A_2b_ and A_3_ (Fredholm et al. 2001). Whereas the activation of A_2_ and A_3_ facilitates nociception (Sawynok 1998), the trigger of A_1_ spinal receptors further slows responses of C fibre-mediated pain transmission in the neurons of the dorsal horn which subsequently causes anti-nociception. Again, the anti-nociceptive effect of A_1_ receptor agonist is also mediated through adenylate cyclase inhibition and a sealing of ATP-sensitive K^+^ Channels. From this study, the second phase of anti-nociceptive activity of EthE was significantly reversed by the adenosine antagonist, theophylline. This effect may be due to a surge in endogenous adenosine release by the extract and/or the extract’s ability to activate A_1_ receptors. The possible involvement of ATP potassium channel in the anti-nociceptive effect of EthE was further highlighted through pretreatment of mice with glibenclamide (an ATP-sensitive K^+^ channel antagonist) before they were given EthE. The results indicated that only the late phase was significantly reversed by the extract indicating the involvement of ATP-dependent potassium channels.

Though there is ample evidence that indicates the involvement of muscarinic, adrenergic, calcium channels and serotonergic pathways in nociception, the results obtained revealed that their respective antagonists could not significantly reverse the anti-nociceptive effect of the extract. Results from the acute toxicity study did not show any toxic effect that could be attributed to drug treatment. The LD_50_ of EthE after oral administration to mice could be estimated to be above 5000 mg/kg. This indicates that EthE is relatively nontoxic based on the recommendations of the Organization for Economic Co-operation and Development (OECD) for chemical labelling and classification of acute systemic toxicity based on oral LD_50_ values.

## Conclusions

EthE inhibits hypernociception induced by TNF-α, IL-1β, bradykinin and prostaglandin E_2_. EthE exhibited anti-nociceptive effects possibly mediated through opioidergic, adenosinergic, ATP-sensitive potassium channels and nitric oxide cyclic GMP pathways.
